# Posttranslational Modification Defects in Fibroblast Growth Factor Receptor 1 as a Reason for Normosmic Isolated Hypogonadotropic Hypogonadism

**DOI:** 10.1155/2020/2358719

**Published:** 2020-11-21

**Authors:** Hui Ying, Yan Sun, Huixiao Wu, Wenyu Jia, Qingbo Guan, Zhao He, Ling Gao, Jiajun Zhao, Yiming Ji, Guimei Li, Chao Xu

**Affiliations:** ^1^Department of Endocrinology and Metabolism, Shandong Provincial Hospital Affiliated to Shandong First Medical University, 324, Jing 5 Road, Jinan, 250021 Shandong, China; ^2^Department of Endocrinology and Metabolism, Shandong Provincial Hospital Affiliated to Shandong University, Jinan, China; ^3^Institute of Endocrinology, Shandong Academy of Clinical Medicine, Jinan, China; ^4^Shandong Provincial Key Laboratory of Endocrinology and Lipid Metabolism, Jinan, China; ^5^Department of Pediatrics, Shandong Provincial Hospital Affiliated to Shandong First Medical University, Jinan, China; ^6^Department of Endocrinology, Qingdao Municipal Hospital, Qingdao, China; ^7^Scientific Center, Shandong Provincial Hospital Affiliated to Shandong First Medical University, Jinan, China

## Abstract

Some mutations in *FGFR1* affect the sense of smell while others do not, resulting in Kallmann syndrome (KS) and normosmic isolated hypogonadotropic hypogonadism (nIHH), respectively. The underlying mechanism is still unclear. *FGFR1* variants are found in less than 10% of patients with KS and nIHH, and among them, only some have undergone functional analysis. Thus, the correlation between the phenotype and genotype cannot be clearly verified. This study reports a case of nIHH and explores the potential mechanism of the *FGFR1* gene in the pathogenesis of nIHH. A preschooler with cryptorchidism, micropenis, strabismus, and hypopsia is described. As he had a normal sense of smell, he was diagnosed with nIHH. A de novo mutation in *FGFR1* (c.2008G>A) was detected in the patient along with a novel variant in *CEP290* (c.964G>A) inherited from his mother. We present compelling in vitro evidence that this *FGFR1* mutation-induced posttranslational modification defect, including defective glycosylation and impaired *trans*-autophosphorylation, along with the final reduction in expression, could lead to impairment of the receptor and abnormal signaling and eventually result in developmental abnormalities and inhibition of GnRH neuron release. The identification of an additional variant suggests that *CEP290* might play a potential role in GnRH development.

## 1. Introduction

Fibroblast growth factor receptor 1 (*FGFR1*), a receptor tyrosine kinase encoded by the gene *FGFR1*, plays a critical role in the formation, survival, and migration of neurons [1]. It is particularly essential for the neurons that control the secretions of downstream sex hormones by producing gonadotropin-releasing hormone (GnRH), which affects sexual development before birth or during puberty [2]. A dominant-negative FGFR1 mutant targeted to GnRH neurons resulted in a 30% reduction in the GnRH neuron number and led to reproductive defects in mice [3]. Additionally, in the process of neuron migration, mutations in *FGFR1* have also been identified to have an influence on olfactory function by affecting the migration route shared with olfactory sensory neurons (ORNs) [4, 5].

Although the expression of *FGFR1* has been found in the nasal placode and in developing olfactory bulbs [6, 7], it is still unclear why some variants in *FGFR1* affect the sense of smell (resulting in Kallmann syndrome (KS)) [8] while others do not (leading to normosmic isolated hypogonadotropic hypogonadism (nIHH) [9]. In addition, a few mutations recurring at the same residue across KS and nIHH [10] make it even more elusive for explaining the pathogenesis, and thus the high degree of heterogeneity induced by *FGFR1* mutations has become a hot research area.

Acting as a basic member of the fibroblast growth factor receptor family, *FGFR1* is composed of three immunoglobulin- (Ig-) like domains in the extracellular domain, which contains a single transmembrane region and a cytoplasmic domain encompassing two types of catalytic receptor tyrosine kinase (TK) domains [11, 12]. Cellular responses are elicited by binding to FGF8 [13] and heparin sulfate proteoglycans (HSPG), resulting in *FGFR1* dimerization, *trans*-autophosphorylation, and activation. Then, four major intracellular signaling pathways, namely, the phosphatidylinositide 3-kinase/AKT (PI3K/AKT), the mitogen-activated protein kinase (MAPK/ERK), phospholipase C (PLC) *γ*, and JAK/STAT pathways, could be activated [14–16]. Additionally, as an N-glycosylated protein, glycosylation of *FGFR1* is important for proper folding during protein maturation, and only with correct subcellular localization and protein trafficking could this receptor be able to bind to its specific ligands and be activated [17, 18].

To date, mutations in *FGFR1* account for less than 10% of patients with congenital hypogonadotropic hypogonadism (CHH) involving KS and nIHH [19], and among them, even fewer have undergone functional analysis, and thus, the correlation between phenotype and genotype cannot be clearly verified. Here, we describe a case of a nIHH patient carrying two variants in distinct candidate genes, *FGFR1* and *CEP290*. In addition to predicting that *CEP290* could be a new candidate gene for CHH, the biochemical significance of the *FGFR1* mutant in vitro was identified for the first time.

## 2. Materials and Methods

### 2.1. Study Subject

This study was conducted according to the guidelines referring to human research as stated in the Declaration of Helsinki. The Institutional Review Board approved the research protocol, and written informed consent was obtained from the parents of the participant. All family members received specific physical and laboratory examinations in Shandong Provincial Hospital. Peripheral blood specimens were collected from each member for genetic analysis. Their olfactory function was tested by the UPSIT Test (University of Pennsylvania Smell Identification Test) or by exposure to different concentrations of volatile solutions.

### 2.2. Mutation Analysis

Genomic DNA was extracted from peripheral blood leucocytes sampled from all the members using a genomic DNA kit (Tiangen Biotech, Beijing, China, DP304-03). The whole-exome sequencing (WES) was performed on DNA from peripheral blood. After the processes of fragmenting the genomic DNA, ligating the paired-end adaptor, amplifying, and purifying, all of the human exons and the 50 bp bases in their adjacent introns were captured by the SeqCap EZ MedExome Target Enrichment Kit (Roche NimbleGen). The DNA library was used to perform postcapture amplification and purification, and then DNA was sequenced by the Illumina HiSeq sequencing platform. Sequence data alignments to the human genome reference (hg19) and variant-calling were used by the NextGENe V2.3.4 software to further get the coverage and mean read depth of the target regions. The mean read depth was 151.24x, and it even reached 20x for 97.95% of the target sequences. Sanger sequencing was used to verify the underlying variant spotted by WES as previously described [20]. The sequence variations were aligned with the reference sequence of *FGFR1* (GenBank accession NM_023110.2) and *CEP290* (GenBank accession NM_025114.3). Genes and proteins are described according to the Human Genome Variation Society (HGVS) nomenclature guideline.

### 2.3. Bioinformatic Assays

To test whether the mutation was benign or malignant, we used software such as PolyPhen-2 (http://genetics.bwh.harvard.edu/pph2/) and Mutation Taster (http://www.mutationtaster.org/) to predict potential effect [21, 22]. Multiple sequence alignment was performed by using Clustal Omega (https://www.ebi.ac.uk/Tools/msa/clustalo/) [23], and the degree of sequence conservation was visualized through Jalview (Version: 2.10.2) and WebLogo (http://weblogo.threeplusone.com/create.cgi). Silico predictions of subcellular *FGFR1* localization were performed by WoLF PSORT (https://wolfpsort.hgc.jp/). A protein-protein interaction network was made by using STRING (version: 11.0) (https://string-db.org/cgi/input.pl?sessionId=z2x8t6A7HFgq&input_page_show_search=on). A tertiary structure model of the TK domain of the wildtype *FGFR1* was download from PDB (PDB ID: 5A46.1A), while the spatial structure of the mutant *FGFR1* protein was predicted from the Swiss model server (https://www.swissmodel.expasy.org/) [24]. All the models were presented on the PyMOL software (version 1.3).

### 2.4. Functional Analysis

#### 2.4.1. Plasmid Construction of FGFR1 and Cell Transfection

Wildtype (WT) *FGFR1* was generated by PCR mutagenesis of a cDNA encoding FGFR1 as previously described [25]. Mutant *FGFR1* (c.2008G>A, p. E670K) was created by the QuickChange Site-Directed Mutagenesis Kit (Stratagene, La Jolla, USA) according to the manufacturer's instructions. WT and mutant *FGFR1* cDNA were, respectively, subcloned into pcDNA3.1+ (Invitrogen, Carlsbad, USA). The sequences of the plasmids were confirmed by Sanger sequencing, and then they were each transiently transfected into HEK (293) cells using Lipofectamine 3000 (L3000-015, Invitrogen, USA). Whole cell lysates were collected for RNA and protein extraction 48 h after transfection. For RNA extraction, the cells were harvested using TRIzol followed by RNAiso plus (Takara, China) according to the manufacturer's instructions. RNA was then converted to cDNA using the PrimeScript™ RT Master Mix (Takara, China). The quantitative PCRs were prepared with SYBR Green (DBI® Bioscience) and were performed in a Roche LightCycler 480 detection system (the primer sequences are shown in the supplementary materials (available here)). In terms of protein levels, after lysing the cells using RIPA buffer with proteinase inhibitors (Invitrogen), the proteins were separated with 8% SDS-polyacrylamide electrophoresis after denaturation and then analyzed by western blotting.

#### 2.4.2. Immunofluorescence

Forty-eight hours after transfection, cells were grown on glass coverslips, and cell culture dishes were fixed with 4% paraformaldehyde, permeabilized with 0.5% Triton X-100, and blocked for 1 h in 5% FBS. Immunostaining was accomplished with anti-FGFR1 (1 : 200; Abcam, Shanghai, China) overnight at 4°C. Species-specific Alexa Fluor 555 secondary antibodies (Invitrogen, Waltham, MA) were used at 1 : 500 at room temperature for 1 h. Nuclei were visualized by DAPI (4′6-diamidino-2-phenylindole, blue). Protein localization was observed by fluorescence microscopy (Carl Zeiss, Germany).

#### 2.4.3. Deglycosylation Analysis

For Endo H digestion, WT and the altered FGFR1 proteins in the whole cell extracts were boiled for 10 min in 10x denaturation buffer (5% SDS, 10% *β*-mercaptoethanol) and incubated with Endo H for 1 h at 37°C according to the manufacturer's recommendations (New England Biolabs). For the PNGase F digestion, the cell extracts were first incubated with 5x Rapid PNGase F Buffer for 5 min at 75°C, then reacted with PNGase F for 10 min at 50°C according to the manufacturer's recommendations (New England Biolabs). Digestion products were fractionated on SDS-PAGE and subjected to immunoblot analyses as described above.

#### 2.4.4. Signal Pathway Assays

After transfection for 24 h, cells were placed in medium without serum for another 24 h, and then incubated for 15 min with 1 nM FGF8 (PeproTech Inc., USA). Subsequently, the cells were subjected to immunoblotting with anti-p-FGFR1 (1 : 5000, Abcam), anti-FGFR1 (1 : 3500, Abcam), anti-p-ERK (1 : 1000, Cell Signaling Technology), anti-ERK1/2 (1 : 1000, Cell Signaling Technology), anti-p-Akt (1 : 1000, Cell Signaling Technology), anti-Akt (1 : 1000, Cell Signaling Technology), anti-p-STAT3 (1 : 1000, Cell Signaling Technology), anti-STAT3 (1 : 1000, Cell Signaling Technology), and anti-GAPDH (1 : 7000, Proteintech) to observe the expression of the FGFR1 phosphorylation levels as well as the relative phosphorylation levels of the downstream signaling molecules.

## 3. Results

### 3.1. Clinical Characteristics

A five-year-old preschooler with cryptorchidism and micropenis was enrolled in our research. As an assisted fertility baby, he was first diagnosed with hypogonadotropic hypogonadism with a small penis of a length of 0.5 cm and a diameter of 0.5 cm at about nine months old. His testicles were detected in the inguinal region with a volume of 0.8 × 0.4 × 0.6cm (left) (testicularvolume(TV) < 1ml) and 0.7 × 0.4 × 0.7cm (right) (TV < 1ml) by ultrasound, together with an empty scrotum. No obvious abnormality was found in intelligence or height. In the follow-up period, the proband was verified with a normal sense of smell, together with a strong argument for CHH in differential diagnosis [26] (Supplementary Table 1), and he was finally diagnosed with nIHH at the age of five. In addition, compared with the phenotypes of the previously reported KS patients [27–29] (Table 1), it can easily be seen that our patient had only a minor phenotype with neither anosmia, skeletal dysplasia, nor hearing loss, but with abnormalities in sexual development.

Moreover, the results of laboratory tests (shown in Table 2) revealed a decreased level of follicle-stimulating hormone (FSH), luteinizing hormone (LH), and testosterone. After performing a GnRH stimulation test by triptorelin acetate injection, the right testicle gradually descended into the right scrotum, and the levels of LH and FSH were upregulated significantly compared to the base value (Supplementary Table 2). No abnormality was found in the other biochemical tests.

Additional auxiliary examinations such as MRI of the pituitary gland showed no significant changes in terms of its shape. All of these data above were harmonious with the primary diagnosis. Notably, the proband had abnormalities in the pursuit test and had slight strabismus as well. In addition, a gradual diminution of vision was found since the initial diagnosis. No ocular abnormality was detected in his parents.

### 3.2. Genetic Analysis

**Figure 1:**
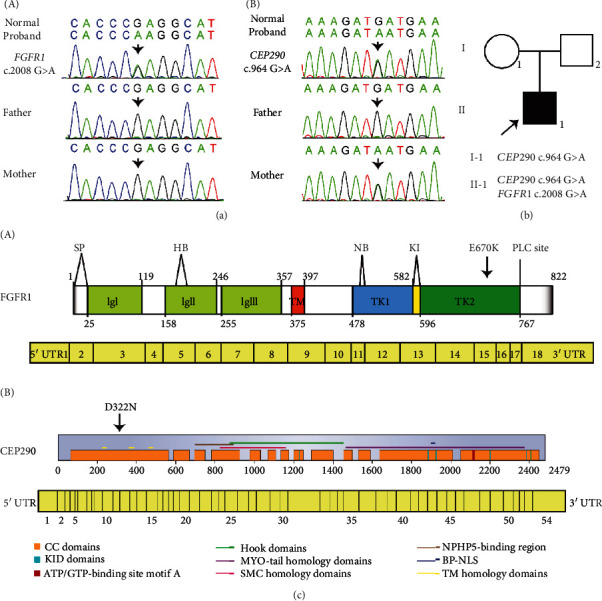
Pedigrees of the proband with *FGFR1* and *CEP290* mutation. (a) The mutation results in both *FGFR1* (A) and *CEP290* (B) from all family members. Arrows: mutation sites. (b) Pedigrees of the proband. Circles: females; squares: males; arrows: proband. (c) The domain structures of both FGFR1 (A) and CEP290 (B) with two mutant sites, respectively. SP: signal peptide; HB: binding domain for heparin or heparin sulfate proteoglycan; PLC sites: interaction with PLC gamma; NB: nuclear-binding domain; TK1/2: tyrosine kinase subdomain 1/2; Ig I, Ig II, and Ig III: three Ig-like domains; TM: transmembrane domain.

To further confirm the primary diagnosis, the whole exome sequencing was performed in all of the available family members. The proband not only harbored a de novo heterozygous mutation in *FGFR1* (c.2008G>A, p. E670K) but also carried a novel heterozygous c.964G>A (p. D322N) *CEP290* variant inherited from his mother, and no variants were detected in his father (Table 1 and Figure 1). The variant c.964G>A in *CEP290* was not identified in the database of the Genome Aggregation Database (gnomAD) (http://gnomad.broadinstitute.org), the Human Gene Mutation Database (HGMD) (http://www.hgmd.cf.ac.uk/), or in a panel of 100 normal Chinese controls.

### 3.3. Bioinformatic Analysis

**Figure 2:**
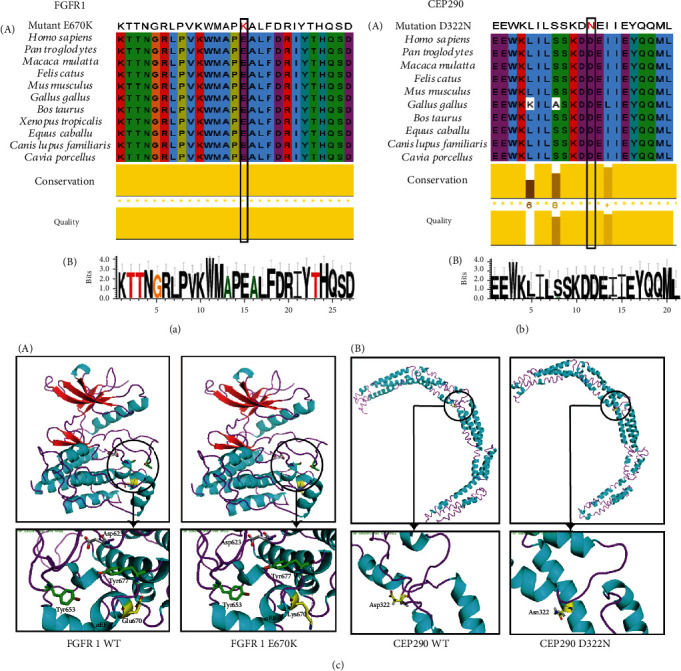
The bioinformatic assays of the mutations of *FGFR1* and *CEP290*. (a, b) (A) Conservation analysis of the two mutant sites via multiple sequence alignment. Amino acid in red color are the substitutive amino acid. Asterisks represent a high score of conservation degree. (B) The conservation degree of FGFR1 and CEP290 calculated by WebLogo software. The overall stack height represents the sequence conservation at that position, while the symbol height within the stack indicates the relative frequency of each amino or nucleic acid at that position. (c) The crystal structure of the mutation E670K upon the catalytic (tyrosine kinase) cytosolic domain of FGFR1 and the maps of the coiled-coil domain within mutant and WT CEP290. *α*-Helices, *β*-strands, and loops are colored cyan, red, and pink, respectively. Nitrogen and oxygen are colored blue and red, respectively. The mutation sites are labeled with yellow sticks. The phosphorylation site of Tyr-653 and Tyr-638 are shown as green sticks. The activation site is shown as grey sticks. Arrows point to the magnified pictures of selected residues. These structural images are shown using PyMOL.

Since both of the variants were located in the structural domain of the coding regions, we speculated that they might have pathogenic significance. Using in silico analysis, we evaluated the disease-causing potential of these substitutions and found that both of the missense variants affected highly conserved amino acids in diverse biological species by multiple sequence alignment. Concurrently, the high score of the conservation degree in both *FGFR1* and *CEP290* calculated by Jalview and WebLogo software (Figures 2(a) and 2(b)) suggested that these mutations were in highly conserved domains across species, and they probably had a disruptive influence on the structure of the proteins. Moreover, the predictions of Mutation Taster and PolyPhen-2 (shown in Supplementary Figure 1) also revealed that the sequence alterations in the conserved residues probably affected protein features as well as splice sites, which were considered to have a disease-causing effect.

When compared with the maps of the TK domain in the WT and mutant FGFR1, Glu-670, situated in the helix of *α*EF, was very close to the activation loop containing the tyrosine autophosphorylation site Tyr-653 and Tyr-677 and the active site Asp-623 (Figure 2(c), A). The carbonyl oxygen of Glu-670 could engage in an intramolecular hydrogen bond with the backbone nitrogen of Tyr-677, and then substitution with lysine would disrupt the hydrogen bond interaction. Furthermore, the E670K substitution changed the charge of the amino acid from negative to positive, thus impairing hydrogen bonds as well as leading to an abnormal charge distribution and structural perturbations. In addition, the partial structure of the coiled-coil domain in the C-terminal lobe of *CEP290* was predicted to present with a significant conversion between a helix and a coil by a 3D model from I-TASSER (Figure 2(c), B), and the mutated D322 residue changed the secondary structure from a coil to an *α*-helix. The overall structure of the coiled-coil domain became more compact in the mutant *CEP290* compared with that in the WT.

Given that the FGFR1 mutation was predicted to change a lot on the structure, we additionally performed silico predictions of subcellular localization to detect whether the variant blocked the normal receptor-ligand binding. Unluckily, the results suggested no difference between the wildtype and the variant (Supplementary Figure 2) to see that both of them probably localized to the plasma membrane; the FGFR1 E670K mutation was thus predicted unlikely to affect protein localization.

### 3.4. Functional Analysis

**Figure 3:**
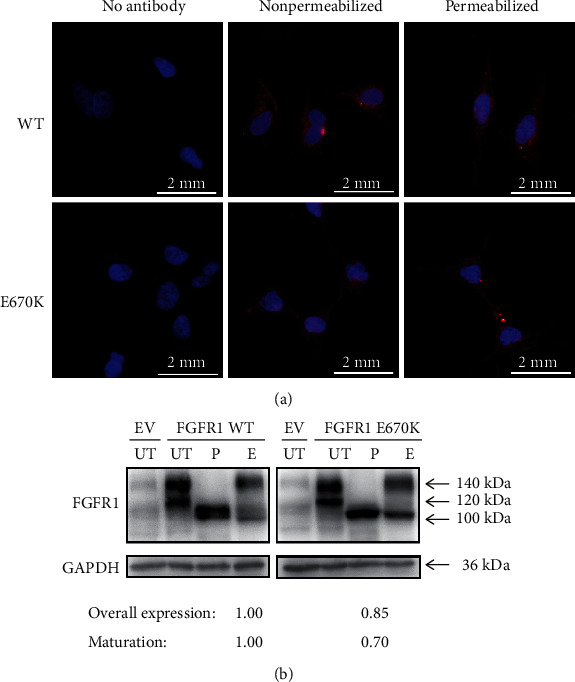
Analysis of subcellular localization and deglycosylation in FGFR1 groups. (a) Subcellular localization of FGFR1 in HEK293 cells. HEK293 cells were transfected with empty vector (EV), FGFR1 (WT), or mutant FGFR1 (p. E670K), and protein localization was observed by fluorescence microscopy. FGFR1 was detected using an anti-FGFR1 antibody followed by secondary antibodies conjugated with Alexa Fluor 555 (red). Nuclei were visualized by DAPI. Original magnification: 600x. (b) Deglycosylation in FGFR1 groups with Endo H and PNGase F. Overall expression levels of FGFR1 in distinct groups were judged from the PNGase F treatments and were normalized to their GAPDH levels, respectively. Maturation analysis was determined from the Endo H-treated groups; the upper band represents the fully glycosylated mature form while the lower band stands for an immature or a core glycosylated form. Percentage of the mature band by density calculations was used to measure the maturation degrees with the groups. Both the results represent the ratio between mutant and WT. EV: empty vector; UT: untreated; E: Endo H-treated; P: PNGase F treated. Arrows point to the molecular weight.

To identify the predicted consequences of the *FGFR1* missense mutation, which has not been confirmed by experimental tests in previous studies, we performed a series of functional analysis. The first we tested was an immunofluorescence assay for subcellular localization identification, and the results showed that both WT and the mutant could be detected in permeabilized cells as well as in nonpermeabilized cells, mirroring that there was no difference between the wildtype and the mutant, consistent with the silico analysis above (Figure 3(a)).

Subsequently, we tested the overall expression levels of WT and mutant FGFR1 via western blotting and performed a deglycosylation experiment for receptor maturation analysis with two distinct glycosylases, PNGase F and Endo H. As shown in Figure 3(b), two immune-reactive-specific bands at 140 kDa and 120 kDa could be detected in the two groups, representing a complete form and a core glycosylation form, respectively. When treated with PNGase F, which could remove almost all N-linked oligosaccharides from the glycoproteins, the two bands in both groups were digested to a single band of ~100 kDa. The overall expression of the E670K mutant in the PNGase F-treated group decreased to approximately 85% compared to WT.

In contrast, when processed with Endo H, which could release only high mannose N-linked sugars and affect only the partially processed receptor (120 kDa) to retain the fully glycosylated mature form (140 kDa), more than half of the wildtype FGFR1 remained at 140 kDa representing a fully glycosylated mature form, whereas only 37% of the mutant protein was resistant to Endo H, indicating that most of the E670K mutant protein presented as a core glycosylated form. When comparing the maturation levels of the groups, densitometric analysis revealed that the E670K substitution only reached 70% of the WT group, which was consistent with the in silico predictions of destabilizing its spatial structure.

**Figure 4:**
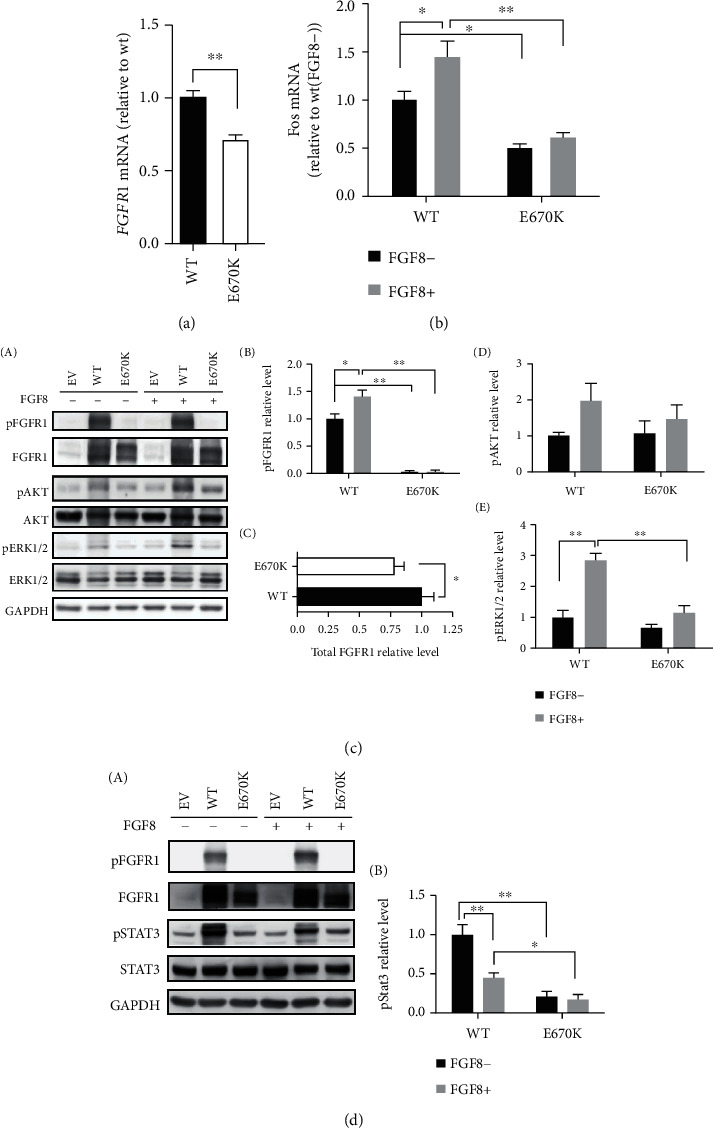
Functional analysis of FGFR1 in the mutant and WT groups in vitro. (a) Gene expression analysis of FGFR1 in the mutant and WT groups by qPCR. HEK293 cells were transiently transfected with WT, mutant FGFR1 plasmid, or empty vector for RNA extraction, using RT-PCR and real-time quantitative PCR to detect the FGFR1 mRNA expression. (b–d) Gene and cell surface expression analysis of *FGFR1* and its downstream signaling. (b) Using RT-PCR and qPCR for the *FOS* gene expression analysis in the FGF8-induced mutant and WT groups. (c) The phosphorylation levels of WT and mutant FGFR1 and the affected signal pathways tested by western blotting in groups (A). Quantitative analyses of FGFR1 phosphorylation levels (Y653) (B), the total FGFR1 relative level (C), FGF8-induced ERK1/2 (D), and Akt (E) phosphorylation levels are shown with a bar chart. (d) Analysis on the FGFR1-affected JAK/STAT3 pathways by western blotting in groups (A). Quantitative analysis of the STAT3 phosphorylation levels (B). Shown is the meanpercentage ± SD of three biological replicates (*p* < 0.01 by Student's *t*-test).

An impaired glycosylation level made us speculate whether the structural instability could lead to protein degradation and finally affect the protein expression. To confirm this, we measured *FGFR1* mRNA expression of the WT and mutant groups, finding that the mRNA expression of the mutant FGFR1 was reduced to ~75% of the level seen for the WT (*p* < 0.01) (Figure 4(a)). Since the mutation in *FGFR1* could partially suppress gene transcription, we additionally speculated that its downstream pathways might also be affected.

Given that c-*FOS*, an important transcription factor downstream of ERK1/2 signaling (one of the essential downstream pathways of FGFR1), is crucial for the activation of the GnRH neurons as well as the synthesis and secretion of FSH, we tested the mRNA level of its coding gene, *FOS*, after activating FGFR1 with its specific ligand FGF8. It has been demonstrated that the activation of FGFR1 has a cascade effect. When the ligand binds to its receptor, dimerization as well as autophosphorylation of the receptor will occur first. The *FOS* expression level showed a dramatic decrease of ~50% in the mutant group in the presence of FGF8 (Figure 4(b)), which is to be expected for the mutant receptor being not activated after binding to the ligand.

In addition to the protein glycosylation level during posttranslational modification, phosphorylation levels of FGFR1 have an influence on receptor activation as well. So, we subsequently tested the phosphorylation level of FGFR1 (Figure 4(c), A). The level in the mutant group was close to 0 with no change even after being stimulated with FGF8 (Figure 4(c), B), while in the WT group, there was an ~30% increase after stimulation. At the same time, we also measured the total protein expression in the two groups and found that the results were consistent with that of RNA expression (Figure 4(c), C).

After demonstrating that the E670K substitution did harm the receptor activation and affected the downstream pathways at the mRNA levels, we additionally measured the phosphorylation level of MAPK/ERK signaling as well as PI3K/AKT signaling (Figures 4(c), A, 4(c), D, and 4(c), E). For the PI3K/AKT pathway, the consequence of the variant mainly appeared after stimulation, revealing that the phosphorylation level in the mutant group was only 75% of that seen in the WT group. Consistent with the same trend in the PI3K/AKT pathway, the FGFR1 mutation grossly affected the phosphorylation level of ERK1/12 signaling (Figure 4(c), E).

On the one hand, the phosphorylation level of pERK1/2 in the substitution group was slightly lower than that of the WT control in the FGF8(-) treatments. On the other hand, when the expression level in the WT control was increased nearly 2-fold, the level in the mutant group was only one third that in the WT group after treatment with FGF8 (*p* < 0.01). Hence, our data showed that the mutation inhibited receptor activation and resulted in impaired downstream signaling.

In addition, further experiments on JAK/STAT3 signaling, another downstream pathway of FGFR1 proposed to have functions in sexual development, were conducted to explore any potential effects on the STAT3 pathway. As shown in Figure 4(d), the activated WT receptor inhibited the phosphorylation level of STAT3; however, the inhibitory effect was too weak to be detected in the mutant treatment. In addition, the STAT3 phosphorylation level in the nonactivated WT group was much higher than that in the corresponding mutant group, suggesting that the E670K substitution not only had an effect on the base phosphorylation level of STAT3 but also prevented JAK/STAT3 signaling from being inhibited by affecting the activation of FGFR1.

### 3.5. The Interaction Network between FGFR1 and CEP290

E670K substitution in FGFR1 has been demonstrated to lead to CHH by our vitro experiment, but the other gene mutation detected in the proband showed no exact evidence to have a correlation with CHH from previous studies except for a silico prediction to be pathogenic in the present study.

By searching the GeneCards database, we found that the gene *CEP290* is encoding for a centrosomal protein 290 (NP_079390), which is a large centrosomal protein of 290 kDa with several motifs that are highly conserved throughout evolution (Figure 1). *CEP290* localizes to the connecting cilium, the transitional zone linking the inner and outer segments of rods and cones, and has been demonstrated to have an important role in ciliary trafficking and cilium assembly [30, 31].

**Figure 5:**
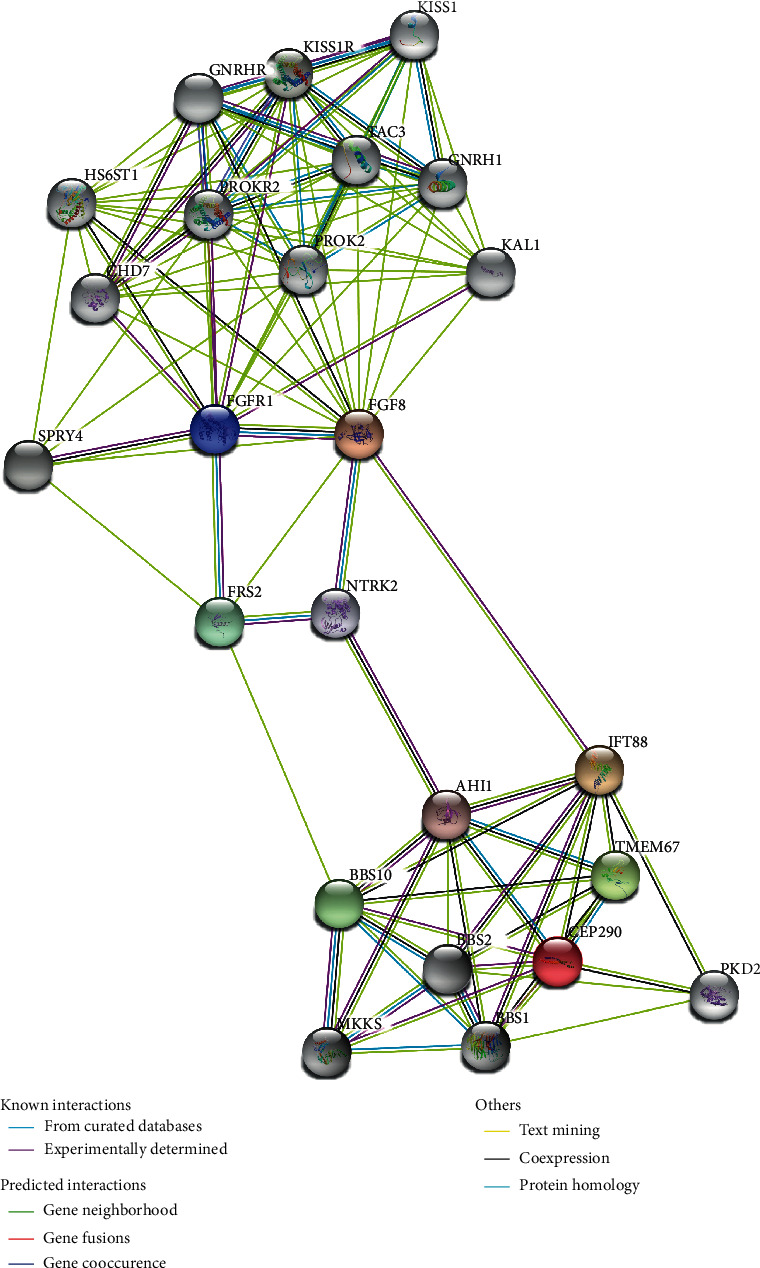
A relevance between GnRH-deficiency-related signaling network and several known disease-causing genes of olfactory dysfunction by using STRING software. The protein-protein interaction network encoded by pathogenic genes known to cause KS including FGFR1 (colored blue) were correlated with CEP290-related protein-protein network involving olfactory dysfunction via several pathways containing the factors of FGF8, NTRK2, AHI1, and IFT88.

Given that studies have verified that *CEP290* was involved in the formation of primary cilia of the olfactory epithelium (OE) [32], which was identified to be an essential step within the GnRH neuron migration route, we thus speculated that *CEP290* might have an interaction with FGFR1 or shared a common pathway in a specific time or region. Subsequently, a protein network was made by using STRING software and surprisingly, there existed an indirect correlation between them indeed (Figure 5). From the protein-protein interaction network encoded by pathogenic genes known to cause KS, we could find that FGFR1 could be correlated with *CEP290*-related protein network involving olfactory dysfunction via the factors of FGF8, NTRK2, AHI1, and IFT88, while pathways with these signal molecules above are crucial on cell cycle, neuron proliferation, cell differentiation, apoptosis, the regulation of gene expression, etc. The results above indicated that *CEP290* probably has a correlation with FGFR1-related network.

## 4. Discussion

Herein, we confirmed a de novo mutation (c.2008G>A) in *FGFR1* for the first time from a nIHH proband by in vitro experiments, and we additionally detected a novel heterozygous variant from *CEP290*, one of the pathogenic genes of ciliopathies identified to have a crucial role in the development of the olfactory epithelium [31]. We presented compelling evidence in vitro that these *FGFR1* mutation-induced posttranslational modification defects, combined with the reduction in both RNA and protein levels, lead to an impaired receptor and abnormal signaling, and eventually resulted in development abnormalities and GnRH neuron-releasing inhibition. Thus, the E670K substitution in *FGFR1* represented a loss-of-function protein.

The phenotype of our proband was relatively mild compared with that in the KS subjects reported previously [28]. Of note, the KS proband carried an additional *FLRT3* gene defect, which was also identified to be associated with CHH and that occurred in KS probands. We therefore speculated that the presence of multiple disease-related genes in one person might synergize their effects to produce a superposition effect to varying degrees, leading to a relatively severe phenotype similar to that of the additive loss of GnRH neurons in Fgfr1^+/-^ Fgf8^+/-^ double heterozygous mice and Il17rd and Spry2 synergy in zebrafish [33, 34]. Furthermore, variable expressivity [8], to some extent, could in part account for the phenotype discrepancy between Asian and Caucasian patients. Unfortunately, we could not obtain more details of the clinical manifestations from the two other reported cases in the paper; therefore, the comparison was only with one case. To further confirm this, more relevant cases should be collected for phenotype-genotype correlation studies.

From the results of bioinformatic and functional experiments, the FGFR1 variant destroyed the structural stability of the protein and induced degradation of the unstable protein. Together with its haploinsufficiency (heterozygosity), the expression in the FGFR1 mutant subject ultimately manifested a significant reduction when compared with the wildtype control. Another remarkable consequence of this mutation was defective posttranslational modifications, involving an entirely impaired phosphorylation level and incomplete glycosylation of the receptor, which were responsible for the receptor being inactivated.

Numerous genes have been identified to be associated with nIHH, but less than 40% of the patients are reported to harbor mutations and are considered to have hereditary defects [35–37], emphasizing the fact that there are many unknown candidate genes associated with nIHH. In the present study, we detected an additional *CEP290* mutation from the patient. This gene was mainly identified as one causative gene for ciliopathies [38], and it is especially critical for OSNs (multiciliate cells) [39]. Recessive mutations in *CEP290* can cause diverse phenotypes, ranging from isolated blindness, olfactory dysfunction in Leber Congenital Amaurosis (LCA), nephronophthisis (NPHP), Joubert syndrome (and related disorders) (JS (RD)), and Bardet-Biedl syndrome (BBS), to the lethal Meckel-Gruber syndrome (MKS) [31]. It is worth noting that in patients with LCA or MKS, aside from the abnormal sense of smell caused by the *CEP290*-induced olfactory epithelium dysplasia, GnRH deficiency to varying degrees can also be detected, because the olfactory epithelium is involved in the first stage of the neuronal migration, and if the epithelium is impaired, the GnRH neurons would not reach their final residence, the hypothalamus [40]. Combined with FGFR1-*CEP290* interaction network results, we speculated that the gene *CEP290* was a candidate gene related to KS.

Although the proband only manifested some eye-related problems rather than an abnormal sense of smell, which we explained by incomplete penetrance, the potential effect of the variant on the migration of GnRH neurons is still worthy of study. Unfortunately, we were unable to construct an overexpression plasmid of the *CEP290* protein for functional analysis of the mutant *CEP290* because of its massive molecular weight, but mouse models with a certain *CEP290* mutation have provided valuable evidence for its possible role in cell division and in maintenance of kidney and retinal homeostasis and in neuronal development during development [41]. In addition, *CEP290* was extensively distributed in embryonic tissue and in different cancerous tissues, which resembles FGFR1, suggesting that they might be involved in similar work during embryonic development or might have some overlaps in clinical features when mutations happened, and both of them were critical in cell survival and function.

KS and nIHH were originally classified as two distinct entities within CHH, but recent reports found that overlaps do exist between the candidate genes of these two entities [28]. For example, the same variant in one family could lead to either anosmia or a normal sense of smell [10], which was also slightly different from the observation in our study. All of these findings, in conjunction with the high degree of heterogeneity of the phenotypic manifestations presented in these pedigrees with multiple pathogenetic gene variants and the emerging idea of oligogenic inheritance [42], support the view that CHH should be considered as a complex genetic disorder that is characterized by variable expressivity and penetrance [36, 43]. Multiple gene variants, environmental factors, or epigenetic modifications may contribute to its variable disease manifestations.

In summary, since most CHH patients have no clear molecular basis so far, new strategies and advanced methods are required to bridge this gap. On such a basis, our study thus provides compelling evidence for elucidating the molecular mechanisms of the mutant FGFR1, while the detection of the *CEP290* substitution suggests a new candidate gene for oligogenic inheritance.

## Figures and Tables

**Table 1:** The correlation of genotypes and phenotypes between proband nIHH and reported Kallmann patient.

Proband	Our research	Previously reported
Dx	nIHH	KS
Sex/age of onset (month)	M/1	F/U
Clinical phenotype		
Puberty	+	+
Anosmia	—	+
Skeletal dysplasia	—	+
Hearing loss	—	+
Dental agenesis	—	—
Strabism	+	U
Left palate	—	U
Genetic mutation analysis		
Mutation	*FGFR1* (E670K)	*FGFR1* (E670K)
	*CEP290* (D322N)	*FLRT3* (Q69K)
Genotype	Heterozygous	Heterozygous
Mutation type	Missense	Missense
Reference	De novo	[28]

Abbreviations are as follows: KS—Kallmann syndrome; Dx—diagnosis; S—sporadic; F—familial; “+”—abnormal (absent puberty is defined as testicularvolume ≤ 3ml or primary amenorrhea at presentation); “U”—unknown; “-”—normal; E—glutamic acid; K—lysine; D—aspartic acid; N—asparagine; Q—glutamine.

**Table 2:** The results of auxiliary examination on proband nIHH.

Clinical tests	Results (reference range)
Laboratory results	
FSH	0.96 (1.27-19.26 mIU/ml)
LH	0.39 (1.24-8.62 mIU/ml)
Testosterone	0.01 ng/ml
Prolactin	12.67 (2.64-13.13 ng/ml)
GnRH stimulation test	Exaggerated LH response to GnRH stimulation
MRI examination of pituitary gland	Normal
Ultrasound test of reproductive system	Bilateral hypoechoic inguinal nodules, cryptorchidism, and hypoplasia of testis

Abbreviations are as follows: nIHH—normosmic idiopathic hypogonadotropic hypogonadism; FSH—follicle-stimulating hormone; LH—luteinizing hormone; GnRH—gonadotropin-releasing hormone; MRI—magnetic resonance imaging.

## Data Availability

All data included in this study are available upon request by contacting the corresponding author.

## Supplementary Materials

Supplementary Materials Supplementary Table 1: differential diagnosis between CHH and constitutional delay of growth and puberty. Supplementary Table 2: the results of a gonadotropin-releasing hormone stimulation test on the proband. Supplementary Figure 1: Mutation Taster as well as PolyPhen-2 predictions on the mutations of FGFR1 and *CEP290*. Supplementary Figure 2: silico predictions of subcellular localization on the mutation of FGFR1 by WoLF PSORT.
