# Supplementary material for: Posttranslational Modification Defects in Fibroblast Growth Factor Receptor 1 as a Reason for Normosmic Isolated Hypogonadotropic Hypogonadism
Source: Oxid Med Cell Longev. 2020 Nov 21;2020:2358719. doi: 10.1155/2020/2358719 (PMC7704206; doi:10.1155/2020/2358719)

**Post-translational Modification** **Defects in Fibroblast Growth Factor Receptor 1 as a Reason for Normosmic Isolated** **Hypogonadotropic** **Hypogonadism**

**Hui Ying^1,2,3,4^,** **Yan Sun^5^,** **Huixiao Wu^1,2,3,4^, Wenyu Jia^6^,** **Qingbo Guan^1,2,3,4^,** **Zhao He^3,4^,** **Ling Gao^1,2,4,7^,** **Jiajun Zhao^1,2,3,4^,** **Yiming Ji^1,2,3,4^, Guimei Li^5^, Chao Xu ^1,2,3,4^***

^1^ Department of Endocrinology and Metabolism, Shandong Provincial Hospital Affiliated to Shandong First Medical University, 324, Jing 5 Road, Jinan 250021, Shandong, China;

^2^ Department of Endocrinology and Metabolism, Shandong Provincial Hospital affiliated to Shandong University, Jinan, China;

^3^ Institute of Endocrinology, Shandong Academy of Clinical Medicine, Jinan, China;

^4^ Shandong Provincial Key Laboratory of Endocrinology and Lipid Metabolism, Jinan, China;

^5^ Department of Pediatrics, Shandong Provincial Hospital Affiliated to Shandong First Medical University, Jinan, China

^6^ Department of Endocrinology, Qingdao Municipal Hospital, Qingdao, China

^7^ Scientific Center, Shandong Provincial Hospital affiliated to Shandong First Medical University, Jinan, China.

***Correspondence:**

Dr. Chao Xu, M.D., PhD.

E-mail: [doctorxuchao@163.com](mailto:doctorxuchao@163.com)

Tel: +86-53168776375

Fax: +86-531-87068707.

**Supplementary Materials**

**Supplementary Table 1.** Differential diagnosis between CHH and constitutional delay of growth and puberty.

| Differential points | CHH | CDGP |
| --- | --- | --- |
| Family history | Just a few (<30%) have a positive family of CDGP | a positive family history of CDGP more frequently (>63%) |
| Clinical features | CHH-associated phenotypes, like cryptorchidism (>30%) or Micropenis, congenital anosmia (i.e., unrelated to facial trauma, surgery, or chemical exposure), cleft defect, synkinesia, renal agenesis, etc. | Less than 2% have a history of cryptorchidism |
| Auxiliary examinations（before puberty） | TV <1ml;  INB <60pg/l;  LH<4.3 IU/L after GnRH-stimulated | TV ≥1.1ml;  INB≥60pg/l;  LH≥4.3 IU/L after GnRH-stimulated |
| Genetic testing | Mutations in at least one CHH gene were found in 51% of CHH probands, which is significantly higher than in CDGP (7%) or controls (18%). Similarly, oligogenicity (defined as mutations in more than one gene) was common in CHH patients (15%) relative to CDGP (1.4%) and controls (2%). | Most of them do not have CHH-related gene mutations (see the left) |
| Treatment | GnRH deficiency is permanent, hormonal treatment is always in need | CDGP is a state of transient GnRH deficiency where puberty eventually begins and is completed without hormonal treatment |

Abbreviations are as follows: CHH, congenital hypogonadotropic hypogonadism; Constitutional delay of growth and puberty, CDGP; Inhibin B, INB; Testicular volume, TV; FSH, Follicle-Stimulating Hormone; LH, luteinizing hormone.

**Supplementary Table 2.** The results of Gonadotropin-releasing hormone stimulation test on the proband.

| Time (min) | 0 | 30 | 60 |
| --- | --- | --- | --- |
| FSH (mIU/ml) | 0.96 | 10.56 | 11.22 |
| LH (mIU/ml) | 0.39 | 2.12 | 2.02 |

Abbreviations are as follows: FSH, Follicle-Stimulating Hormone; LH, luteinizing hormone.

**Supplementary Figure 1.** Mutation Taster as well as PolyPhen-2 predictions on the mutations of FGFR1 and CEP290.


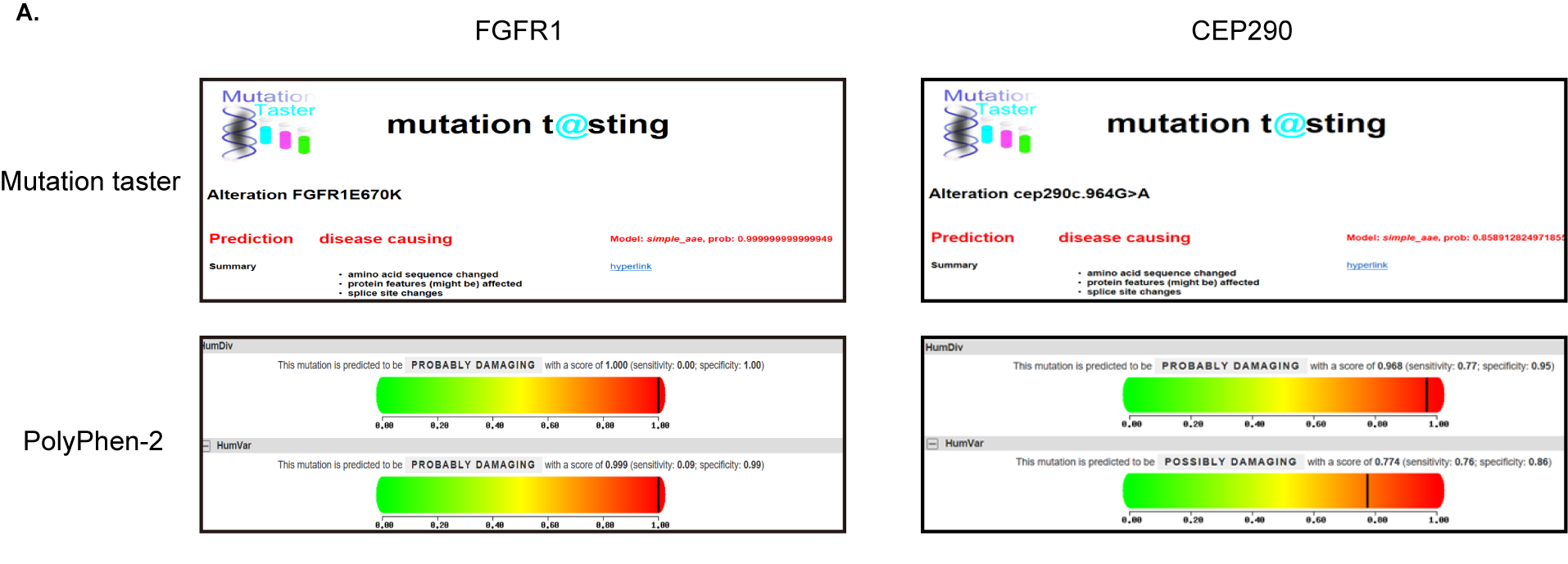


**Supplementary Figure 2.** Silico predictions of subcellular localization on the mutation of FGFR1 by WoLF PSORT.


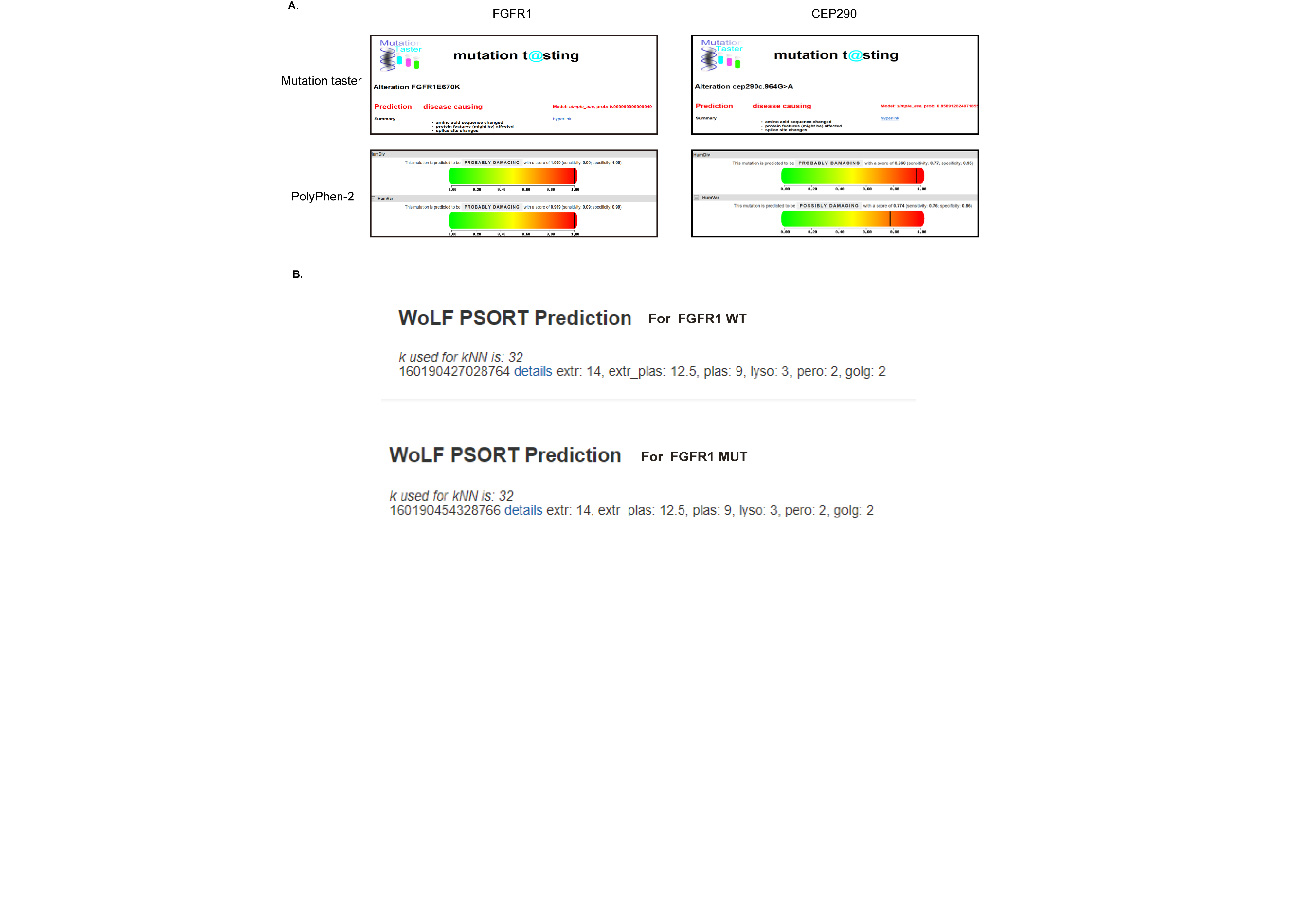

Supplement: Supplementary Materials — Supplementary Table 1: differential diagnosis between CHH and constitutional delay of growth and puberty. Supplementary Table 2: the results of a gonadotropin-releasing hormone stimulation test on the proband. Supplementary Figure 1: Mutation Taster as well as PolyPhen-2 predictions on the mutations of FGFR1 and CEP290. Supplementary Figure 2: silico predictions of subcellular localization on the mutation of FGFR1 by WoLF PSORT. [file 2358719.f1.doc]
